# Co-Operation in a Contributory Scheme

**Published:** 1922-10

**Authors:** L. S. Luckham

**Affiliations:** President of the Salisbury and District Infirmary and Hospital League


*To the Editor of* THE HOSPITAL AND HEALTH REVIEW.

### Co-operation in a Contributory Scheme.

*To the Editor of* THE HOSPITAL AND HEALTH REVIEW.

SIR,—In your September issue you published a letter in which I gave some particulars of our contributory scheme. I should like to add that, at a conference of hospital representatives held at the Salisbury General Infirmary, the following resolutions were put forward as a basis for further consideration :

1. That a contributory scheme should be adopted throughout the area served by their hospitals where such does not already exist.
2. That the general hospitals concerned should be asked to agree upon boundaries.
3. That in each such hospital district the cottage hospitals be asked to form a branch under the name of that hospital, *e.g.*, the Salisbury and District Infirmary and Hospital League—Andover Branch.
4. That all contributory members should have their *maintenance* contribution paid for them when an inmate of any hospital or convalescent home.
5. That the balance of funds available afterwards should at the end of every three or six months be allocated as follows :—
   - (*a*) To each hospital the total average cost of every such member using that hospital—less the maintenance already paid.
   - (*b*) Any further balance to be divided on the same scale according to the number of patients.

The hospitals represented were the Royal Hampshire County Hospital, Winchester ; the Royal South Hants and Southampton Hospital, Southampton ; the Westminster Memorial Hospital, Shaftesbury ; the Romsey Nursing Home and Cottage Hospital ; the Andover Cottage Hospital ; the Warminster Cottage Hospital ; and the Fordingbridge Cottage Hospital.

The report on page 358 (September issue) of the meeting between the Voluntary Hospitals Commission and Hospital Committees, and especially the remarks of Mr. Crouch (Somerset), show the absolute necessity of some fair co-operative system of collecting in all those areas where general and cottage hospitals overlap, and where hospitals draw patients from other counties. We think our proposals will meet this difficulty and tend to make some regular contributory scheme almost universal. We have gone into the figures as to other cottage-hospital districts, and find that in all the figures support my contention that they do not meet their share of hospital expenditure.

Yours faithfully,

L. S. LUCKHAM,

President of the Salisbury and District Infirmary and Hospital League.

